# Supplementary material for: MetaQTL: a package of new computational methods for the meta-analysis of QTL mapping experiments
Source: BMC Bioinformatics. 2007 Feb 8;8:49. doi: 10.1186/1471-2105-8-49 (PMC1808479; doi:10.1186/1471-2105-8-49)
Supplement: Additional File 7 — MetaQTL Package : jar file and tutorial. This Zip archive contains both the MetaQTL JAR file and the files of the tutorial. [file 1471-2105-8-49-S7.zip › org.inra.metaqtl/doc/org/metaqtl/bio/entity/bean/PropertyBean.html]

PropertyBean


|  |  |  |
| --- | --- | --- |
| **Overview** \| **Package** \| **Class** \| **Use** \| **Tree** \| **Deprecated** \| **Index** \| **Help** | |  |
| **PREV CLASS**   NEXT CLASS | **FRAMES**    **NO FRAMES**     **All Classes** |  |
| SUMMARY: NESTED \| FIELD \| CONSTR \| METHOD | DETAIL: FIELD \| CONSTR \| METHOD |  |


---


## org.metaqtl.bio.entity.bean Class PropertyBean

```
java.lang.Object
  org.metaqtl.bio.entity.bean.PropertyBean
```

---

``` public class PropertyBean extends java.lang.Object ```

Class Description Here

**Author:**
:   Jean-Baptiste Veyrieras

---

| **Field Summary** | |
| --- | --- |
| `java.lang.String` | `name` |
| `java.lang.String` | `value` |


**Constructor Summary**

`PropertyBean()`

`PropertyBean(java.lang.String key,
java.lang.String value)`


| **Method Summary** | |
| --- | --- |
| `java.lang.String` | `getName()` |
| `java.lang.String` | `getValue()` |
| `void` | `setName(java.lang.String string)` |
| `void` | `setValue(java.lang.String string)` |

**Methods inherited from class java.lang.Object**

`clone, equals, finalize, getClass, hashCode, notify, notifyAll, toString, wait, wait, wait`

**Field Detail**

### name

```
public java.lang.String name
```

---


### value

```
public java.lang.String value
```


**Constructor Detail**

### PropertyBean

```
public PropertyBean()
```

---


### PropertyBean

```
public PropertyBean(java.lang.String key,
                    java.lang.String value)
```

**Parameters:**: `key` - the key name of the property: `value` - the value of the property


**Method Detail**

### getName

```
public java.lang.String getName()
```

:   **Returns:**

---


### getValue

```
public java.lang.String getValue()
```

:   **Returns:**

---


### setName

```
public void setName(java.lang.String string)
```

:   **Parameters:**: `string` -

---


### setValue

```
public void setValue(java.lang.String string)
```

:   **Parameters:**: `string` -


---


|  |  |  |
| --- | --- | --- |
| **Overview** \| **Package** \| **Class** \| **Use** \| **Tree** \| **Deprecated** \| **Index** \| **Help** | |  |
| **PREV CLASS**   NEXT CLASS | **FRAMES**    **NO FRAMES**     **All Classes** |  |
| SUMMARY: NESTED \| FIELD \| CONSTR \| METHOD | DETAIL: FIELD \| CONSTR \| METHOD |  |


---
